# Bodies out of control: Relapse and worsening of eating disorders in pregnancy

**DOI:** 10.3389/fpsyg.2022.986217

**Published:** 2022-09-28

**Authors:** Bente Sommerfeldt, Finn Skårderud, Ingela Lundin Kvalem, Kjersti S. Gulliksen, Arne Holte

**Affiliations:** ^1^Institute of Eating Disorders, Oslo, Norway; ^2^Department of Psychology, Faculty of Social Sciences, University of Oslo, Oslo, Norway; ^3^Faculty of Health Sciences, University of Southern Denmark, Odense, Denmark; ^4^Faculty of Health and Sports Sciences, University of Agder, Kristiansand, Norway; ^5^The Norwegian Psychological Association, Oslo, Norway; ^6^Norwegian Institute of Public Health (NIPH), Oslo, Norway

**Keywords:** eating disorders, anorexia nervosa, bulimia nervosa, pregnancy, relapse, triggers, risk factors

## Abstract

**Background:**

Being pregnant is a vulnerable period for women with a history of eating disorders. A central issue in eating disorders is searching control of one’s body and food preferences. Pregnancy implies being increasingly out of control of this. Treatment and targeted prevention start with the patient’s experience. Little is known about how women with a history of eating disorder experience being pregnant.

**Method:**

We interviewed 24 women with a history of eating disorder at the time of pregnancy, recruited from five public pregnancy controls at local family health centers in Norway. Interviews were analyzed by means of ideal type analysis, with a particular focus on how the participants experienced pregnancy and perceived triggers in possible experiences of relapse or worsening during pregnancy. All participants completed the Eating Disorder Examination Questionnaire (EDE-Q) and were diagnosed (DSM-5) by using the Eating Disorder Examination (EDE).

**Results:**

On becoming pregnant, 23 of the 24 participants experienced worsening or relapse of their disorder. This occurred both at first time and after several pregnancies, and either interviewed early or late in pregnancy. Ideal type analyses indicated seven different personal features associated with worsening or relapse: the “chaotic” “rigid” “perfect” “worried” “shameful” “motherhood fearing” and “the mother with lost identity” Perceived triggers of worsening or relapse were: “loss of control” “unpredictability” “competition” “change of appearance” “shame and nausea” and “loss of identity.”

**Conclusion:**

Pregnancy is a vulnerable period for women with a history of eating disorders. A great variation in personal psychological dynamics seems to interact with perceived triggers in worsening or relapse of eating disorder. Our findings have important implications in understanding mechanisms of relapse in pregnancy, preventing relapse and help tailoring adequate intervention.

## Introduction

Eating disorders in pregnancy is associated with poor outcomes for both the mother and her baby, including miscarriage, preterm delivery, and fetal disabilities such as poor fetal growth or malformations (Watson et al., 2017; Janas-Kozik et al., 2021). The overall aim of this study is to understand how women with a history of eating disorder may experience pregnancy.

For most people, having an eating disorder mean being emotionally overwhelmed by their body, weight, and appearance. This in turn, is linked to unhealthy behaviors, such as starvation, overeating, vomiting and/or excessive physical activity. The woman may feel controlled by thoughts and feelings about body and food. One side of the disorder is the behavior itself, another will often be the experience of feeling obsessed and of being trapped in compulsive arithmetic details such as calories, grams, kilograms, kilometers, number of repetitions and so on.

A central feature of eating disorder pathology is difficulties in interpreting, regulating, and expressing emotions, and that the body is given a central role in attempts at regulating emotions (Bruch, 1978; Fox and Froom, 2009; Robinson et al., 2019). Lack of inner security and weakened self-regulation often contribute to feeling inadequate, unsuccessful and without satisfactory control (Robinson et al., 2019). The symptoms can be seen as potentially relieving, as strategies that are more or less successful in regaining coherence, vitality and self-regulation (Bruch, 1978; Goodsitt, 1997; Robinson et al., 2019).

Eating disorders are commonly linked to cognitive and emotional preoccupancy with control, including control of one’s own body (Robinson et al., 2019). Being pregnant, on the other hand, is a period of life when the woman is increasingly out of control of her own body. Her body changes without her being able to do much about it. Also, to some extent the woman’s eating preferences, thoughts, and feelings may change and being experienced as beyond control. In addition, she needs to deal with emotional, psychological and relational changes accompanying pregnancy and becoming a mother.

It’s a widespread belief that eating disorders are less common during pregnancy (Morgan et al., 1999; Blais et al., 2000; Bulik et al., 2007; Crow et al., 2008; Micali et al., 2009). This may not be the case. Mental health problems in general seems to increase during this phase of life (Eberhard-Gran et al., 2014; Hahn-Holbrook et al., 2018). A number of studies also exists, which may indicate that pregnancy is a particularly vulnerable period for women with an eating disorder (Claydon et al., 2018; Fogarty et al., 2018; Makino et al., 2020).

The prevalence of eating disorders during pregnancy is rapported being between 5% and 8% (Easter et al., 2013; Pettersson et al., 2016). Some studies have found high incidence of symptomatic relapse in women with an eating disorder (Sollid et al., 2004; Crow et al., 2008; Coker et al., 2013; Makino et al., 2020; Janas-Kozik et al., 2021). Ward (2008) showed that some women develop eating disorders for the first time during pregnancy, whereas others with a prior history can relapse.

A number of studies have rapported that women with a history of eating disorders may find it difficult to adjust to their changing body, and that eating disordered behaviors from previous years may return (Mitchell-Gieleghem et al., 2002; Koubaa et al., 2015; Fogarty et al., 2018). Others have shown that women with a history of eating disorders are extraordinary challenged in the first period of pregnancy (Sollid et al., 2004; Taborelli et al., 2016).

Claydon et al. (2018) rapported that pregnancy and the transition to motherhood can be an extremely challenging time for women with an eating disorder both psychologically and physically. In some women, stress around pregnancy and facing parenthood may seem to produce an uncontrollable urge to restrict weight gain (Patel et al., 2005).

Currently, limited evidence exists on how women with a history of eating disorders themselves experience pregnancy. Studies about relapse in women with a history of eating disorders are sparse. Many of these studies are retrospective (Morgan et al., 1999; Taborelli et al., 2016), questionnaire-based (Sollid et al., 2004; Crow et al., 2008; Makino et al., 2020), unclear in identifying diagnosis (Ward, 2008; Makino et al., 2020), and/or use clinical hospital samples (Taborelli et al., 2016). Such studies may be valuable in their own right, but are also likely to suffer from recall bias, selection bias, or, as in some questionnaire studies, only give quantifiable, sometimes relatively superficial, answers of limited clinical value.

Therefore, we have studied ways in which women with a history of eating disorder may experience being pregnant. The results are presented as a limited number of ideal types based on analyses of comprehensive in-depth interviews with a non-clinical sample of 24 women while they were pregnant. We address the following research questions:

How do women with a history of eating disorders experience being pregnant? How do they understand and describe worsening, relapse and perceived triggers in such eventual changes?

## Materials and methods

### Participants

This study is part of a larger study, “Mummiebodies” In Mummiebodies, we have comprehensively in-depth-interviewed 24 women, first while they were pregnant, then at post-partum. Inclusion criteria were that the participants had a self-rapported history of an eating disorder, had been in treatment for a diagnosed eating disorder within the past 10 years, and were pregnant at the time point of the first interview. Exclusion criteria were any psychotic symptoms.

### Setting and procedure

The participants were recruited from the general population through the universal, public routine pregnancy controls at five local family health care centers in the greater Oslo area in Norway. These services are free. Participation rate at the pregnancy controls at these centers is 93–96%.

Midwives and nurses at the health centers were thoroughly informed about the project, inclusion and exclusion criteria, and asked to invite potential participants. Social media, podcasts and seminars were used to encourage and support the recruitment of participants.

Potential participants sent an e-mail to the first author and were then informed further about the study by e-mail, accompanied by a written invitation to participate. The written invitation contained a detailed description of the research project, again including its purposes and procedures. No one withdrew after having consented to give their contact information to the researcher.

After approving participation, the participant and the interviewer set up a time point to meet. Then, a semi-open qualitative research interview was conducted about how they, having a history of an eating disorder, experienced being pregnant. After the interview, all participants filled out the Eating Disorder Examination Questionnaire, EDE-Q (Bohn and Fairburn, 2008). Finally, Eating Disorder Examination, EDE, was administered to reflect current DSM-5 diagnosis (American Psychiatric Association, 2013).

The aim of the research interview was to provide rich descriptions as precise and as close to the participants’ experiences as possible. Data were collected using the “Experience Interview” (Holte, 2000), a semi-open, participant-centered, strategic conversation format developed from communication theory (Littlejohn, 1999). Rather than using questions, participants were given open instructions such as: “In your own words, please tell me about your pregnancy…” To facilitate the interviewer’s own development of the themes, the interviewer responded mainly by means of continuators (“Tell me more…,” “Go on…”), references to her own impressions (“That sounds hard!”), and frequent use of verbal (“Really!”), and nonverbal (nodding) facilitators and of headlines and structurers (“Now we have talked a lot about…., let us now move on to…”). Use of questions was either open (“Can you describe it more for me?”) and used to elicit further information (“Could you give me an example?), but primarily limited to elicit specific information (“Can you show me how you touch your tummy?”). The interviewer could also make comments on the here-and-now to get the informant to reflect on what was being described (“How is it to tell this to me here-and-now?) or to elicit more emotional information (“You are crying…”).

All interviews were conducted by the first author (BS). Each qualitative interview lasted between 90 and 120 min and was audiotaped and transcribed verbatim by the first author (BS). Altogether, introductory information, interview, and administration of EDE-Q and EDE took three to three and a half hours. All procedures were conducted in accordance with the Helsinki declaration, and the study was approved by the Norwegian Regional Committee for Medical Research Ethics.

### Data analyses

We have used ideal type analyses (Stapley et al., 2021) to give structure to the richness and diversity of this large data material. Typical ways that women with a history of eating disorder may experience pregnancy are organized into multiple ideal types. The method was originally devised by Max Weber to explain sociologically individual cultural phenomena (Weber, 1978).

Ideal type analysis is a branch of interpretive social research dealing with textual data materials that are organized into empirically constructed models. The final product, the ideal types, illuminates the variation and patterns that can exist across the participants, while at the same time showing personal variations between them.

Each ideal type contains personal features and processed statement observed in several participants. In this way, the ideal type approach is positioned between a cross-case approach and a case-study approach (Ayres et al., 2003). Even though the main purpose is to illustrate different types, the types need not to be mutually exclusive. Ideal types are constructed models, not real cases.

The ideal type analyses were carried out in several steps (Stapley et al., 2021): Firstly, the first author (BS) became familiar with the dataset by conducting the interviews, listening through the audio files, transcribing the interviews verbatim, checking accuracy of the transcripts, and reading and re-reading the transcripts. Concurrently, the second (FS) and fifth (AH) authors listened to tape records and were included as co-readers of transcripts and as discussants about possible interpretations.

To get an overall impression and capture relevant and different experiences, the first author made detailed notes from the interview experience and thoughts and comments of potential significance. All the way through, we reflected upon possible biases and preconceived ideas that we might have brought to the analyses and that could possibly influence our interpretations.

By exploring each text according to the “bottom-up principle” (Seidel and Kelle, 1995; Richards, 2015) different themes emerged. Soon it became clear that all except one (23 of 24) of the participants had experienced the bodily changes as difficult. These 23 participants rapported that their relationship with their bodies and their eating problems had worsened significantly, or that their eating disorder had re-emerged.

Therefore, we decided to limit further analysis to worsening or relapse of the eating problems and perceived triggers. Consequently, although interesting as this case contrasted the other 23 cases, we decided to exclude the participant that did not have these problems from further qualitative analysis.

The second step of the analysis was to write a summary of the worsening or relapse that each participant had experienced, including their perception of possible triggers. This summary contained both the woman’s and the researcher’s perspective. This involved reading the interview transcript several times and listing elements in the transcripts that were relevant to the research question. In this way, each summary was used to identify key issues of the participant’s experience and perspective on her worsening or relapse, including possible triggers.

Thirdly, we used the summaries to form clusters of similar experiences to be used in constructing ideal types (Gerhardt, 1994). We did this by systematically comparing and contrasting each summary with each other. We looked for similarities and differences between experiences about their worsening or relapse and what triggered it. The intention was to identify patterns across the data set. This resulted in 11 clusters of experiences which provided the basis for constructing tentative ideal types. The clusters of experiences were identified through team discussion and agreements.

In the fourth step, we tried to find the simplest solution that at the same time provided optimal qualitative balance between taking best care of the variation between the types, giving the best conceptual clarity, and giving the least overlap between types. This re-organizing resulted in seven types. The seven types were given labels for what we regarded as the most relevant personal features characterizing the worsening or relapse.

The fifth step was to form the final descriptions of the ideal types. In this process, for each ideal type we took as point of departure the summary of the participant that best represented the ideal type. We then modified each summary by deleting experiences that were not associated with the cluster of experiences and included experiences that were associated with the cluster. At the end, we smoothed the resulting summary into being a coherent ideal type.

Finally, to check credibility, the analyses were regularly discussed within the research team to ensure that the ideal types were well represented in the data and vice versa. This way, ideal types and interpretations were continuously challenged, discussed and reassessed.

## Results

### Participants

Background information, diagnoses and a brief history of the participants’ eating disorders are shown in Table 1.

**Table 1:** Details of participants.

	Age (Mean 32.8)	Earlier eating disorder diagnosis^a^	Years of treatment for eating disorder^b^	Diagnosis under pregnancy^c^	Gestations week	Eating disorder psychopathology (EDE) Global Score (mean 3.8)^d^	Re-enganged in treatment during pregnancy
Participant 1		Anorexia nervosa, restrictive type	4	Other specified feeding and eating disorder (OSFED), atypical anorexia nervosa	32	4.45	Yes, not specialized
Participant 2		Anorexia nervosa, restrictive type	8	Other specified feeding and eating disorder (OSFED), atypical anorexia nervosa	19	2.3	Yes, not specialized
Participant 3		Anorexia nervosa, restrictive type	5	Other specified feeding and eating disorder (OSFED), atypical anorexia nervosa	23	5.5	Yes, not specialized
Participant 4		Eating disorder not other specified (EDNOS)	5	Other specified feeding and eating disorder (OSFED), atypical anorexia nervosa	16	3.5	Yes, not specialized
Participant 5		Bulimia nervosa	9	Other specified feeding and eating disorder (OSFED), bulimia nervosa of low frequency	12	3.66	Yes
Participant 6		Anorexia nervosa, restrictive type	15	Other specified feeding and eating disorder (OSFED), atypical anorexia nervosa	12	2.7	Yes
Participant 7		Anorexia nervosa, restrictive type	8	None	37	1.5	No
Participant 8		Eating disorder not other specified (EDNOS)	9	Unspecified Eating disorder	15	1.78	No
Participant 9		Bulimia nervosa	3	Bulimia nervosa (BN) – mild	14	4.4	Third-time mother	Yes
Participant 10		Anorexia nervosa, restrictive type	17	Other specified feeding and eating disorder (OSFED), atypical anorexia nervosa	40	5	Yes, not specialized
Participant 11		Anorexia nervosa, restrictive type	3	Other specified feeding and eating disorder (OSFED), atypical anorexia nervosa	9	4.63	Yes, not specialized
Participant 12		Anorexia nervosa, restrictive type	9	Other specified feeding and eating disorder (OSFED), atypical anorexia nervosa	25	1.6	Third-time mother	Yes, not specialized
Participant 13		Bulimia nervosa	9	Other specified feeding and eating disorder (OSFED), atypical anorexia nervosa	22	4.73	Yes, not specialized
Participant 14		Bulimia nervosa	6	Other specified feeding and eating disorder (OSFED), atypical anorexia nervosa	16	3.16	Yes
Participant 15		Anorexia nervosa, restrictive type	7	Other specified feeding and eating disorder (OSFED), atypical anorexia nervosa	23	3	Yes
Participant 16		Anorexia nervosa, restrictive type	11	Other specified feeding and eating disorder (OSFED), bulimia nervosa of low frequency	25	2.44	Yes, not specialized
Participant 17		Bulimia nervosa	3	Bulimia Nervosa (BN) -severe	26	5	Yes, not specialized
Participant 18		Bulimia nervosa	16	Other specified feeding and eating disorder (OSFED), Atypical Anorexia nervosa	17	5.1	Yes
Participant 19		Anorexia nervosa, binging type	3	Unspecified eating disorder	16	5.1	Yes, not specialized
Participant 20		Anorexia nervosa, restrictive type	9	Other specified feeding and eating disorder (OSFED), atypical anorexia nervosa	37	3.66	Yes
Participant 21		Bulimia nervosa	7	Other specified feeding and eating disorder (OSFED), atypical anorexia nervosa	32	2.93	Yes, not specialized
Participant 22		Anorexia nervosa, restrictive type	6	Other specified feeding and eating disorder (OSFED), atypical anorexia nervosa	17	3.17	Yes, not specialized
Participant 23		Anorexia nervosa, restrictive type	2	Other specified feeding and eating disorder (OSFED), atypical anorexia nervosa	27	4.4	Yes
Participant 24		Bulimia nervosa	10	Other specified feeding and eating disorder (OSFED), bulimia nervosa of low frequency	26	5.6	Yes

a Earlier diagnosis is self-rapported based on DSM-IV diagnosis.

b Years of treatment are self-rapported by mail after the interview.

c Diagnosis based in DSM-5 criteria.

d Self-rapport on symptoms using EDE-Q.

The 24 participating women were 26–42 years of age (mean 32.8 years). Age at first-time mothers in Norway is 30.6 years (2021). All information about the participants’ history of eating disorder is based on self-report. All participants rapported to have been in treatment for a diagnosed eating disorder within the past 10 years. None were in specialized treatment for their eating disorder when they became pregnant. Twenty-two rapported in the interview that they were in need of treatment again during pregnancy. Nine of the participants re-started specialized treatment for eating disorder while they were pregnant, 13 rapported that they had asked for treatment for their eating problems during pregnancy at the birth controls, but did not get specialized treatment, 2 did not report the need of specialized treatment in pregnancy. Rapported duration of treatment for the eating disorder varied from 2 years to 17 years (mean 7.7 years). Thirteen were first-time mothers, 9 were second-time mothers, 2 were third-time mothers. Gestation week at the time of the interview varied between week 9 through 40.

Altogether, 23 of the 24 participants qualified for an EDE-assessed DSM 5-diagnosis of eating disorder at the time of the interview. Table 1 shows that 3 qualified for Bulimia Nervosa (BN), 2 for Unspecified Eating Disorder (UFED), 18 for Other Specified Feeding or Eating Disorders (OSFED) and 1 did not qualified for an eating disorder at the time point of interview. OSFED replaces the former Eating Disorder Not Otherwise Specified (EDNOS) category in DSM-IV (American Psychiatric Association, 1994). The table also shows that the symptom pressure measured with EDE-Q Global Score was generally high, varied between 1.6 and 5.6. One of this did not have a diagnosis at the time of the interview, and were not included in the study. The mean EDE-Q Global Score for the 23 participants included in the study were 3.8. This shows that there were high levels of symptoms related to body, food and weight.

### Ideal types

The analyses of the 23 narratives resulted in seven different ideal types. The ideal types describe assumingly typical variations within and between participants in the process of worsening or relapse of eating disorders in pregnancy and what they perceive as triggered it. The seven ideal types have been labeled “The chaotic mother” “The rigid mother” “The perfect mother to be” “The worried mother” “The shameful mother” “The mother who fears motherhood” and “The mother with lost identity” Table 2 summarize the personal features and perceived triggers in the ideal types.

**Table 2:** Ideal types, perceived triggers and personal features.

Ideal type	Perceived trigger	Personal feature
“The chaotic mother”	Loss of control	Chaotic and impulsivity
“The rigid mother”	Unpredictability	Rigidity
“The perfect mother to be”	Competition	Perfectionism
“The worried mother”	Changes in appearance	Dependency and insecurity
“The shameful mother”	Shame and nausea	Shamefulness
“The mother who fears motherhood”	Ambivalence	Motherhood fearing
“The mother with the lost identity”	Loss of identity	Lack of identity

### “The chaotic mother”

Britt (38 years) had been diagnosed with bulimia nervosa, severe type. She had struggled with overeating and vomiting and occasionally self-harm since she was a teenager. She had previously benefited from treatment. The last 2 years had been more stable. She had a partner and a permanent job. Then she got pregnant. With the pregnancy, the bulimic chaos returned to her life.

She experienced being pregnant as a tremendous threat to her sense of control over her life. This included food intake, food preferences, food routines, exercise routines, weight increase, appearance, her thoughts, and her pregnant body in general. “I have lost control of everything,” she said, and “I think about food 24–7” She experienced losing control of her weight gain. She lost control of her food intake and ate very irregularly. She tried to take control of her food and body through old bulimic strategies. During the day she tried to hold back, only to end up overeating and vomiting in the evenings. Vomiting escalated throughout the pregnancy, and this became something that she felt was impossible to stop. “It’s all chaos” she said. Both in thought and emotion and action, she lost control of what was to give her a sense of control. Earlier, she had used overeating and vomiting to cope with difficult feelings. As a pregnant woman, she also tried to eat and “clear away” the fear of becoming a bad mother.

Britt linked the relapse of her bulimia nervosa, severe type, to the *tremendous loss of control* that being pregnant meant to her.

### “The rigid mother”

Lise (32 years) had previously been diagnosed with anorexia nervosa. In her early twenties, she was in treatment for anorexia for a period of time. She had managed to become more flexible in relation to food and exercise, but her weight was still in the lower normal range. She had become pregnant after repeated fertility treatments. She had been told by her mother that she was a very “rigid” child, and that she had been very concerned about predictability. She became very worried about agreements being changed. Lise had also been picky and selective when it came to food ever since she was little. This meant restrictive intake and few “approved” foods.

She experienced being pregnant as a threat to her strong urge for the world to be predictable, her dependency of strict rules, fixed regimens, that agreements were kept, and that no changes in her life would come unexpected. This included her restrictive intake of a modest number of approved foods, weight control, exercise, and social life. “All these changes in pregnancy are too much for me, more than I can bear,” she said.

The pregnancy was filled with obsessions and anxiety. She tried through strict regimes to stop her weight gain during pregnancy. She started counting again: kilos, kilocalories and more. Again, there were a lot of yes-food and no-food” “All the old lists and rules I had before, came back. I’m trying to hold on.”

With her body as a tool, she tried to keep her mind in check. She bought herself a heart rate monitor. She made rules for how many steps she should take daily and how many calories she should allow herself to eat. She could no longer eat at restaurants, and she made all the food herself.

Lisa linked the relapse of her anorectic behavior to the many *unpredictable changes* that the pregnancy meant to her.

### “The perfect mother to be”

Susie (34 years) had struggled with anorexia nervosa in her teens. Ever since childhood, perfectionism – and performance anxiety – had been strong. This had been worked on in therapy. But now she experienced a relapse in pregnancy. She experienced being pregnant as another area to compete on and to become best in. “I’m gonna be the perfect pregnant woman,” she said, “the thin pregnant, have a caesarean section, and be the thin mother” She experienced mastery during pregnancy when she was below the weight curve on the health checks.

Susie was in good physical shape and received a lot of positive feedbacks from the midwife, doctor and family. She received comments that she looked just as slim as before pregnancy. She was proud of this. “I compare myself to pregnant girlfriends and get satisfied when they get bigger and change their body shape.”

“Pregnancy has become something new to compete at. I have to be the perfect pregnant woman.” She followed many pregnant Instagram profiles and compared herself with these, what they ate and how they exercised. She wanted a caesarean section because she wanted to know when she would give birth. She would avoid going over birth term in case of unnecessary fat on her body.

Susie linked the resurgence of her anorectic behavior to the *performance anxiety* that being pregnant and becoming a mother triggered in her.

### “The worried mother”

Tracey (26 years) had previously been diagnosed with anorexia nervosa. Throughout her youth and adulthood, Tracey had felt a strong anxiousness about not being liked and not being good enough. She describes herself as an anxious child. Through the eating disorder in her youth, she had received a lot of attention and confirmation from others. During this second pregnancy, she became more worried about not being liked. She was afraid that her partner would not like her when she was getting bigger and putting on weight. She felt insufficient in this pregnancy. She felt nauseous and unwell the first part of the pregnancy, and was unable to engage in the family life. She became more insecure about herself again during this pregnancy.

Tracey was afraid that this pregnancy would ruin the contact and relationship with her partner. She was afraid that her partner would be more concerned with the child than with her. She had experienced this with her first child.

She experienced being pregnant as a double threat, to her female attraction and confirmation of this, and that her baby should get more attention than herself from her partner, family and friends.

The eating disorder gave her again an experience of mastery and focus. “This pregnancy has made me very self-centered again. I’m obsessed with my own body and trying to control it.” Tracy was “outer-directed” extremely concerned about what her partner thought of her and body. “I ask him several times a day if I’m still attractive.” She was alert to glances and comments from others, both friends and colleagues.

“I was terrified when the midwife said that it was important that I showed interest in the baby in my womb and sensed any differences from the activity of the baby. In all the years with the eating disorder, I have learned to disconnect, and not sense my body.” She did not connect to the baby in her womb. She compared body, weight, and curves with previous pregnancy. She thought a lot about her body, but sensed it to a lesser extent.

Tracy linked the worsening of her eating disordered restrictive behavior to the *changes in appearance* and with that, the *decreased attention and confirmation* that she felt her pregnancy entailed.

### “The shameful mother”

Anna (28 years) was previously diagnosed with bulimia nervosa, severe type. She was pregnant for the second time. She had a difficult childhood with physical and sexual abuse from her stepfather. She had previously used overeating, vomiting and overexercising as ways to forget. But these were also methods for punishing herself. She had been in therapy before she became pregnant for the first time, and her strong feelings of shame was constantly raised.

Anna was ashamed in many ways. She was ashamed of the bad person she meant that she was. She was ashamed of having an eating disorder. She was ashamed of the abuse. And she was ashamed of her body. With the pregnancies came more shame. She experienced being pregnant as shameful over her body, her bulimia nervosa, and her life since she was sexually abused as a child. “I do not feel any joy in this pregnancy. I know it’s wrong.”

Anna had not had overeating episodes or vomited in the last year after she became a mother for the first time. “The bulimia had a break for a few years,” she said.

In her first pregnancy, she had vomited daily until birth. When she had eaten, she had strong negative experiences of her own body. This pregnancy came close to the first one, and she still experienced having a “maternity body where everything was soft and dizzy” She said it felt like she was wearing a big fat suit on the outside of her body.

Anna was angry too. She was angry at her partner. She was angry at the child. And most angry at herself: “I feel a terrible shame about being angry at the child who is destroying my body,” she said. Anna could not bear to touch the pregnant stomach with the palms of her hands. “I tingle and pinch, but find it uncomfortable and disgusting to touch.” With her hand at a distance, she pointed to her stomach. She described how she often scratched herself on the side, “to check how much fat there is” She hated her body even more than before. She avoided showering. She did not dare to appear naked in front of partner, and avoided intimacy. “I probably think that my partner feels the fat on the outside of my body if he hugs me. I cannot do that.” Anna could not walk in clothes that were tight and touched her body. She covered herself with wide clothes. She got some new appetites during pregnancy. She ate what she herself described as “forbidden food” She punished herself by exercising.

Anna linked her relapse to her pregnant body that made her even more shameful. The *nausea and vomiting early in her pregnancy* triggered the urge to vomit after meals. “Once I’ve started vomiting, it’s impossible to stop again,” she said.

### “The mother who fears motherhood”

Sara (26 years) had for many years struggled with anorexia nervosa. She had been told that she would not become pregnant due to long-term anorexia nervosa. She had not regained menstruation even though she had gained normal weight in recent years. The pregnancy came as a shock to her. For her, the eating disorder, she said, was very much about being allowed to be a child without responsibilities and obligations. She was not at all ready to deal with the demands and expectations she associated with being an adult. And now she was to become the adult herself. That scared her.

Sara feared motherhood. She experienced being pregnant as a threat to her wish to continue being a child, that her anorexia nervosa made possible. “I am not ready.” She was close to having an abortion. “I dear not attach myself to the child,” she said, while at the same time dreaming about becoming a mother. This ambivalence created emotional chaos for Sara. She became afraid of eating, lost the balance between exercise and food intake, and was hospitalized midway through her pregnancy.

Sara said she feared she could not take care of both the child and herself and linked her relapse to the *ambivalence between childhood and motherhood and the fear of expectations and responsibility as a mother*.

### “The mother with lost identity”

Philippa (41 years) had for 15 years qualified for the diagnosis of anorexia nervosa. In the last few years, however, it was “atypical anorexia nervosa” Her weight was almost normal. It had been a few years since she had stopped treatment. Philippa experienced her anorexia as a central part of her sense of identity. “Anorexia, that’s who I am. That’s what others perceive me to be as a person” she told. She became very confused throughout her pregnancy. Before, she was described as “the thin and well-trained” “But who am I now?” she wondered several times in despair. “I have lost myself. Now I can no longer measure my efforts and performance through my body.”

Philippa could not exercise as before. Her body could not handle it. She experienced the eating disorder in the form of exercise and healthy food, as something that shaped her and gave her a sense of being someone. It was a project that gave her something concrete to hold on to, even meaning in life. Now she had lost her sense of identity and her life-project. She did not dare to see her own weight with the midwife.

Philippa linked her relapse to a deep *grief over having lost her sense of identity, herself.*

## Discussion

This study describes assumingly typical ways that women who have been in treatment for a diagnosed eating disorder, may experience being pregnant.

Many health professionals seem to believe that eating disorders tend to improve or are less prevalent during pregnancy (Morgan et al., 1999; Blais et al., 2000; Bulik et al., 2007; Crow et al., 2008; Micali et al., 2009). Yet, previous studies have found that these patients have increased risk of relapse (Makino et al., 2020), and that worry about weight may trigger symptoms of eating disorder during pregnancy and the postpartum period (Gerhardt, 1994; Makino et al., 2020).

However, in-depth descriptions of *how* women with a history of eating disorders experience being pregnant has been lacking. Such descriptions are important because any helping relationships to be successful, must take the patient’s experience as it’s point of departure. Our study addresses this gap.

The results show both that there are some common personal features in how women with a history of eating disorders may experience being pregnant, but also that there are a variety of experiences that are unique to the single women.

Most importantly, 23 of 24 of our participants had in common that they experienced worsening or re-emergence of their eating problems when they became pregnant. Our results shows that women with a history of eating disorders in pregnancy report high levels of symptoms during pregnancy, both through self-rapport (EDE-Q) and through diagnostic interview (EDE). This indicates that becoming pregnant is a vulnerability factor for resurgence of symptoms of eating disorder among women with a history of such disorders.

This finding is consistent with previous findings that eating disorders are more prevalent during pregnancy than in other periods of life (Smink et al., 2012; Easter et al., 2013; Pettersson et al., 2016; Hecht et al., 2022), that pregnancy, psychologically as well as physically, can be an extremely challenging time for women with an eating disorder (Claydon et al., 2018), that stress around pregnancy and facing parenthood may produce an uncontrollable urge to restrict weight gain (Patel et al., 2005), that women with a prior history of eating problems may find it difficult to adjust to their changing body (Freizinger et al., 2010), that eating disordered behaviors from previous years may return (Mitchell-Gieleghem et al., 2002; Ward, 2008; Koubaa et al., 2015), and of a high incidence of symptomatic relapse in women with an eating disorder (Sollid et al., 2004; Coker et al., 2013; Makino et al., 2020; Janas-Kozik et al., 2021). Our finding agrees also well with the finding that pregnancy is vulnerable period for women’s mental well-being (Eberhard-Gran et al., 2014; Hahn-Holbrook et al., 2018).

Twenty-two of the 23 women reported in the interview that they were in need of treatment for their eating disorder again. Thirteen of the women did not get any specialized treatment for their eating disorder during pregnancy. This emphasis the need to enhance our competencies to be able to help women in pregnancy.

We also found that worsening of symptoms or relapse of eating disorder could occur both among women who were pregnant for the first time and among women who had been pregnant or given birth before. This may stand in some contrast to previous findings that women with a history of eating disorder are more vulnerable for resurgence of eating problems in their first pregnancy compared to subsequent pregnancies (Taborelli et al., 2016).

Furthermore, we found that women rapported worsening of symptoms or relapse either they were interviewed early in pregnancy (e.g., gestation week nine) or later in pregnancy (e.g., gestation week 40). This may stand in some contrast to others who have found that women with a history of eating disorders are extraordinary challenged in the first period of pregnancy (Sollid et al., 2004; Taborelli et al., 2016).

Our analyses of how women with a history of eating disorder may experience pregnancy, resulted in seven ideal types. We labeled these types according to their personal features: “The chaotic mother” “The rigid mother” “The perfect mother to be” “The worried mother” “The shameful mother” “The mother who fears motherhood” and “The mother with the lost identity” The seven ideal types illustrate what seems to be interactions between the pregnant woman’s personal features and the meaning she attributes to her pregnancy. This again, may seem to trigger relapse or worsening of the eating disorder.

We identified seven such triggers, one or several of which may be involved when a pregnant woman with a history of eating disorder experiences relapse. These were perceiving pregnancy as: a tremendous loss of control that leads to chaos, a large number of unpredictable changes that leads to rigidity, a competitive comparison which leads to competition, perfectionism and performance anxiety, a change in appearance which leads to decreased attention and increased need for confirmation, a shameful body and a course of nausea during early pregnancy which leads to vomiting, a situation of ambivalence between childhood and motherhood which leads to panic and hopelessness, and finally, as a loss of identity which leads to deep confusion.

All the personal features that were challenged when our participants became pregnant are recognizable also among pregnant women with no history of eating disorder. Experiencing a strong need for control, being rigid and requiring that the world is predictable, striving for perfection, seeing the word as a set of areas for competition, being self-centered with a constant need of confirmations, being shameful and self-loathing, not feeling ready for and fearing motherhood, or experiencing that pregnancy changes one’s sense of identity, are all known experiences associated with pregnancy and becoming a mother (Slade et al., 2009).

In most women, these common challenges do not trigger a mental disorder. While, as shown in this study, among women with a history of eating disorder, they may. Critical here, seems to be how the woman attributes meaning to their pregnancy, which again seems to reactivate or to reinforce the eating disorder.

This may indicate that it is not the weight gain, changing body, appetite or body functions in itself that are important when a woman experience worsening of her eating disorder symptoms with pregnancy. It is the meaning that she attributes to such changes, which again seems to interact with her personal features.

This implies both a great diversity in the psychological dynamics behind such relapses, but also a far greater psychological complexity in the relapse than only taking objective measures such as body weight, body change, and changed body functions into consideration. This again may have serious implications for treatment of pregnant women with a history of eating disorder. Speculation about these implications is, however, outside the scope of this study.

Because of the qualitative design in this study, concluding about objective causality, i.e., *x* triggers *y*, is not possible. We may, however, conclude about subjective causality, i.e., *x* is perceived as triggering *y*. Subjective causality may of course correspond to objective causality, or it may not. In this case, we do not know. In this paper, we have therefore only suggested, not ascertained, that what we have called “triggers” actually may trigger worsening or relapse of Anorexia Nervosa, Bulimia Nervosa, or Other Specified Feeding or Eating Disorders. From a therapeutic point of view, however, the patient’s perception of the causal links, subjective causality, may be as important as the real objective causal links. OK.

### Strengths and limitations

Strengths of this study are the non-clinical sample, the comprehensive in-depth interviews supervised by two experienced seniors who are specialized in this particular type of qualitative interview technique, the relatively large number of participating women who all had a history of eating disorder and were pregnant at the interview timepoint, and the analytic procedure, including use of ideal types. The use of standard diagnostic interview while the women were pregnant, strengthens the diagnostic validity of the study.

This study includes eating disorder diagnoses from the DSM-5 (American Psychiatric Association, 2013). In DSM-5 (American Psychiatric Association, 2013) eating disorder is divided into: Anorexia Nervosa (AN), Bulimia Nervosa (BN), Binge Eating Disorder (BED), Other Specified Feeding and Eating Disorders (OSFED) and Unspecified Feeding and Eating Disorders (UFED). None of the participants rapported to have had BED and none received this diagnosis when assessed in our study.

Most previous studies are limited to AN and/or BN. A strength of this study is that we included OSFED and UFED. In DSM-5, OSFED and UFED replaces EDNOS from earlier versions of DSM. Studies show that EDNOS may be as severe and longstanding as one of the specific eating disorders (Fairburn and Beglin, 2008; Qian et al., 2022). OSFED and UFED includes individuals who do not fulfill all the criteria for a specific eating disorder. In this study 18 of 23 participants qualified for OSFED and 2 qualified for UFED. This makes both OSFED and UFED more suitable to pick up pregnant women with a history of eating disorders where clinical characteristics can be temporarily masked during pregnancy. Symptoms of eating disorders such as self-induced vomiting, binge eating and low weight can be more difficult to detect because of typical features of pregnancy.

Eating disorder diagnoses tend to be unstable (Castellini et al., 2011) and may frequently switch between Anorexia Nervosa, Bulimia Nervosa, and Other Specified Feeding and Eating Disorders (OSFED). Therefore, we included all three diagnoses. This has the clinical advantage that attention is concentrated on the processes across diagnostic categories rather than on single diagnoses or single cases. However, this multi-diagnostic approach might also be a limitation because if we had used one diagnostic category only, we could had looked more into variation within one of the three categories.

Another limitation in this study is that we did not recruit women with Binge Eating Disorder and therefore is not included in the study.

One clear limitation of the study is that we do not know how representative the participants are of pregnant women with a history of eating disorder. Therefore, we cannot know whether any of the personal features or the participants’ attribution of meaning to their pregnancy is more prevalent than others, either in the general or in the clinical population.

It is notable, however, that 23 out of 24 participants rapported relapse or worsening of their eating disorder during pregnancy, and that all the 23 participants qualified for one of the three diagnoses of eating disorder while pregnant. This extremely skewed distribution could indicate that reactivating of eating problems while pregnant is highly prevalent among women with a history of eating disorder.

Even though the referring health services were instructed to refer pregnant women with a history of eating disorder, not with a current eating disorder, we cannot exclude that the extremely skewed distribution reflects selection bias. To the referring health service personnel, pregnant women with a current eating disorder may have been easier to detect and to invite than those with a history, but no current eating disorder.

Another limitation is that even though all participants in the analyses qualified for an eating disorder diagnosis while interviewed during pregnancy, we have no knowledge about how many or which one of the participants that were recovered from the diagnoses before they became pregnant. We could therefore not differentiate between women who still had the diagnosis when they got pregnant and then worsened their condition during pregnancy, and those who had recovered and relapsed when they became pregnant.

All participants had been in treatment because of their eating disorder. In all cases this included some kind of psychotherapy. This may have induced both a better understanding and ability to formulate issues related to their personal features as well as how they attributed meaning to their pregnancy. In that case, we may have studied iatrogenic effects and not the participants genuine understanding of how pregnancy triggered relapse or worsening of their eating problems. However, conducting such a relatively large study with participants who have never been in treatment would probably not have been feasible. The assumption of a genuine understanding in terms of not being influenced by others including family members, friends, and colleagues, who would most likely be holding common perceptions of eating disorders, would also hardly be feasible. And if it had been feasible, it would probably had been of little practical value.

### Future research and conclusions

Eating disorders are known to seriously affect pregnancy and pregnancy outcomes. Our study shows the important of knowledge about the experiences of pregnancy in women with a history of eating disorders for preventing relapse. This knowledge can help us to better recognize and tailor help and support for women in a vulnerable and sensitive period in life.

## Data availability statement

The original contributions presented in the study are included in the article/supplementary material, further inquiries can be directed to the corresponding author.

## Ethics statement

The studies involving human participants were reviewed and approved by Regional Committees for Medical and Health Research Ethics (REC) 20th of May 2020, Reference 92665. The participants provided their written informed consent to participate in this study. Written informed consent was obtained from the individual(s) for the publication of any potentially identifiable images or data included in this article.

## Author contributions

BS, FS, and AH contributed to conception and design of the study. BS conducted the interviews, transcribed the interviews and analyzed the material. AH and FS listened to the tape records and were co-readers of transcripts and as discussants about possible interpretations. The analyses were continuously challenged, discussed and reassessed by all authors. BS wrote the first draft of the manuscript. AH and FS wrote sections of the manuscript. All authors contributed to the manuscript revision, read, and approved the submitted version.

## Funding

The research work has been financially supported by the Norwegian Women’s Public Health Association who grant for the project “mummy bodies, eating disorders, pregnancy and postpartum”, Grant: 40404.

## Conflict of interest

The authors declare that the research was conducted in the absence of any commercial or financial relationships that could be construed as a potential conflict of interest.

## Publisher’s note

All claims expressed in this article are solely those of the authors and do not necessarily represent those of their affiliated organizations, or those of the publisher, the editors and the reviewers. Any product that may be evaluated in this article, or claim that may be made by its manufacturer, is not guaranteed or endorsed by the publisher.

## References

[ref1] American Psychiatric Association (1994). Diagnostic and Statistical Manual of Mental Disorders. ed. APA. 5th ed. (Washington, DC: American Psychological Association).

[ref2] American Psychiatric Association (2013). “Diagnostic and statistical manual of mental disorders. Chapter 18,” in Diagnostic and Statistical Manual of Mental Disorders. ed. APA. 5th ed. (Washington, DC: American Psychological Association).

[ref3] Ayres L. Kavanaugh K. Knafl K. A. (2003). Within-case and across-case approaches to qualitative data analysis. Qual. Health Res. 13, 871–883. doi: 10.1177/1049732303013006008, PMID: 12891720

[ref4] Blais M. A. Becker A. E. Burwell R. A. Flores A. T. Nussbaum K. M. Greenwood D. N.. (2000). Pregnancy: outcome and impact on symptomatology in a cohort of eating-disordered women. Int. J. Eat. Disord. 27, 140–149. doi: 10.1002/(SICI)1098-108X(200003)27:2<140::AID-EAT2>3.0.CO;2-E, PMID: 10657887

[ref5] Bohn K. Fairburn C. (2008). Eating Disorders Examination – Questionnaire. Norsk versjon. Regional avdeling for spiseforstyrrelser (RASP). Oslo: Oslo Universitetssykehus HF

[ref6] Bruch H.. (1978). The Golden Cage: The Enigma of Anorexia Nervosa. Cambridge, MA: Harvard University Press.

[ref7] Bulik C. M. Von Holle A. Hamer R. Knoph Berg C. Torgersen L. Magnus P.. (2007). Patterns of remission, continuation and incidence of broadly defined eating disorders during early pregnancy in the Norwegian mother and child cohort study (MoBa). Psychol. Med. 37, 1109–1118. doi: 10.1017/S0033291707000724, PMID: 17493296 PMC2657803

[ref8] Castellini G. Lo Sauro C. Mannucci E. Ravaldi C. Rotella C. M. Faravelli C.. (2011). Diagnostic crossover and outcome predictors in eating disorders according to DSM-IV and DSM-V proposed criteria: a 6-year follow-up study. Psychosom. Med. 73, 270–279. doi: 10.1097/PSY.0b013e31820a1838, PMID: 21257978

[ref9] Claydon E. A. Davidov D. M. Zullig K. J. Lilly C. L. Cottrell L. Zerwas S. C. (2018). Waking up every day in a body that is not yours: a qualitative research inquiry into the intersection between eating disorders and pregnancy. BMC Pregnancy Childbirth 18:463. doi: 10.1186/s12884-018-2105-6, PMID: 30497443 PMC6267071

[ref10] Coker E. L. Mitchell-Wong L. A. Abraham S. F. (2013). Is pregnancy a trigger for recovery from an eating disorder? Acta Obstet. Gynecol. Scand. 92, 1407–1413. doi: 10.1111/aogs.12256, PMID: 24015979

[ref11] Crow S. J. Agras W. S. Crosby R. Halmi K. Mitchell J. E. (2008). Eating disorder symptoms in pregnancy: a prospective study. Int. J. Eat. Disord. 41, 277–279. doi: 10.1002/eat.20496, PMID: 18027861

[ref12] Easter A. Bye A. Taborelli E. Corfield F. Schmidt U. Treasure J.. (2013). Recognising the symptoms: how common are eating disorders in pregnancy? Eur. Eat. Disord. Rev. 21, 340–344. doi: 10.1002/erv.2229, PMID: 23495197

[ref13] Eberhard-Gran M. Slinning K. Rognerud M. (2014). Screening for postnatal depression—a summary of current knowledge. Tidsskr. Nor. Laegeforen. 134, 297–301. doi: 10.4045/tidsskr.13.0068, PMID: 24518477

[ref14] Fairburn C. G. Beglin S. J. (2008). “Eating disorder examination questionnaire (EDE–Q 6.0),” in Cognitive Behavior Therapy and Eating Disorders. ed. Fairburn C. G. (New York: Guilford Press), 309–314.

[ref15] Fogarty S. Elmir R. Hay P. Schmied V. (2018). The experience of women with an eating disorder in the perinatal period: a meta-ethnographic study. BMC Pregnancy Childbirth 18:121. doi: 10.1186/s12884-018-1762-9, PMID: 29720107 PMC5932857

[ref16] Fox J. R. Froom K. (2009). Eating disorders: a basic emotion perspective. Clin. Psychol. Psychother. 16, 328–335. doi: 10.1002/cpp.622, PMID: 19639651

[ref17] Freizinger M. Franko D. L. Dacey M. Okun B. Domar A. D. (2010). The prevalence of eating disorders in infertile women. Fertil. Steril. 93, 72–78. doi: 10.1016/j.fertnstert.2008.09.055, PMID: 19006795

[ref18] Gerhardt U. (1994). What is a case study and what is it good for? Am. Polit. Sci. Rev. 98, 341–354.

[ref19] Goodsitt A. (1997). “Eating disorders: a self-psychological perspective,” in Handbook of Treatment for Eating Disorders. eds. Garner D. M. Garfinkel P. E. (Washington DC: The Guilford Press), 205–228.

[ref20] Hahn-Holbrook J. Cornwell-Hinrichs T. Anaya I. (2018). Economic and health predictors of national postpartum depression prevalence: a systematic review, meta-analysis, and meta-regression of 291 studies from 56 countries. Front. Psych. 8:248. doi: 10.3389/fpsyt.2017.00248, PMID: 29449816 PMC5799244

[ref21] Hecht L. M. Hadwiger A. Patel S. Hecht B. R. Loree A. Ahmedani B. K.. (2022). Disordered eating and eating disorders among women seeking fertility treatment: a systematic review. Arch Womens Ment. Health 25, 21–32. doi: 10.1007/s00737-021-01156-x, PMID: 34175997

[ref22] Holte A. (2000). Serious diagnosis: the patient’s experience. Paper presented at the Sixth International Congress on Behavioral Medicine, Brisbane, Australia.

[ref23] Janas-Kozik M. Żmijowska A. Zasada I. Jelonek I. Cichoń L. Siwiec A.. (2021). Systematic review of literature on eating disorders during pregnancy-risk and consequences for mother and child. Front. Psych. 12:777529. doi: 10.3389/fpsyt.2021.777529, PMID: 34966309 PMC8710601

[ref24] Koubaa S. Hällström T. Brismar K. Hellström P. M. Hirschberg A. L. (2015). Biomarkers of nutrition and stress in pregnant women with a history of eating disorders in relation to head circumference and neurocognitive function of the offspring. BMC Pregnancy Childbirth 15:318. doi: 10.1186/s12884-015-0741-7, PMID: 26613953 PMC4662826

[ref25] Littlejohn S. W. (1999). Theories of Human Communication. Belmont, CA: Wadsworth.

[ref26] Makino M. Yasushi M. Tsutsui S. (2020). The risk of eating disorder relapse during pregnancy and after delivery and postpartum depression among women recovered from eating disorders. BMC Pregnancy Childbirth 20:323. doi: 10.1186/s12884-020-03006-7, PMID: 32460729 PMC7251919

[ref28] Micali N. Simonoff E. Treasure J. (2009). Infant feeding and weight in the first year of life in babies of women with eating disorders. J. Pediatr. 154, 55–60. doi: 10.1016/j.jpeds.2008.07.003, PMID: 18783793

[ref29] Mitchell-Gieleghem A. Mittelstaedt M. E. Bulik C. (2002). Eating disorders and childbearing: concealment and consequences. Birth 29, 182–191. doi: 10.1046/j.1523-536X.2002.00186.x, 12153649

[ref30] Morgan J. F. Lacey J. H. Reid F. (1999). Anorexia nervosa: changes in sexuality during weight restoration. Psychosom. Med. 61, 541–545. doi: 10.1097/00006842-199907000-00019, PMID: 10443763

[ref31] Patel P. Lee J. Wheatcroft R. Barnes J. Stein A. (2005). Concerns about body shape and weight in the *postpartum period and their relation to women’s self-identification*. J. Reprod. Infant Psychol. 23, 347–364. doi: 10.1080/02646830500273657

[ref32] Pettersson C. B. Zandian M. Clinton D. (2016). Eating disorder symptoms pre- and postpartum. Arch Womens Ment. Health 19, 675–680. doi: 10.1007/s00737-016-0619-3, PMID: 26961005

[ref33] Qian J. Wu Y. Liu F. Zhu Y. Jin H. Zhang H.. (2022). An update on the prevalence of eating disorders in the general population: a systematic review and meta-analysis. Eat. Weight Disord. 27, 415–428. doi: 10.1007/s40519-021-01162-z, PMID: 33834377 PMC8933366

[ref34] Richards L. (2015). Handling Qualitative Data. A Practical Guide. London: SAGE Publications Ltd.

[ref35] Robinson P. Skårderud F. Sommerfeldt B. (2019). Hunger. Mentalization-Based Treatments for Eating Disorders. Springer. doi:10.1007/978-3-319-95121-8

[ref36] Seidel J. Kelle U. (1995). “Different functions of coding in the analysis of textual data” in Computer-Dided Qualitative Data Analysis: Theory, Methods, and Practice. ed. Kelle U. (Thousand Oaks, CA: Sage).

[ref37] Slade A. Cohen L. J. Sadler L. S. Miller M. (2009). “The psychology and psychopathology of pregnancy: reorganization and transformation. Chapter 2” in Handbook of Infant Mental Health. ed. Zeanah C. H. Jr. (New York: The Guildford Press), 22–24.

[ref38] Smink F. R. van Hoeken D. Hoek H. W. (2012). Epidemiology of eating disorders: incidence, prevalence and mortality rates. Curr. Psychiatry Rep. 14, 406–414. doi: 10.1007/s11920-012-0282-y, PMID: 22644309 PMC3409365

[ref39] Sollid C. P. Wisborg K. Hjort J. Secher N. J. (2004). Eating disorder that was diagnosed before pregnancy and pregnancy outcome. Am. J. Obstet. Gynecol. 190, 206–210. doi: 10.1016/S0002-9378(03)00900-1, PMID: 14749661

[ref40] Stapley E. OKeeffe S. Midgley N. (2021). Essentials of Ideal-Type Analysis: A Qualitative Approach to Constructing Typologies. Washington, DC: American Psychological Association.

[ref41] Taborelli E. Easter A. Keefe R. Schmidt U. Treasure J. Micali N. (2016). Transition to motherhood in women with eating disorders: a qualitative study. Psychol. Psychother. 89, 308–323. doi: 10.1111/papt.12076, PMID: 26493834

[ref42] Ward V. B. (2008). Eating disorders in pregnancy. BMJ 336, 93–96. doi: 10.1136/bmj.39393.689595.BE, PMID: 18187726 PMC2190274

[ref43] Watson H. J. Zerwas S. Torgersen L. Gustavson K. Diemer E. W. Knudsen G. P.. (2017). Maternal eating disorders and perinatal outcomes: a three-generation study in the Norwegian mother and child cohort study. J. Abnorm. Psychol. 126, 552–564. doi: 10.1037/abn0000241, PMID: 28691845 PMC5505270

[ref44] Weber M. (1978). Economy and Society: An Outline of Interpretive Sociology. Berkeley, CA: University of California Press.

