# Corrigendum: Bodies out of control: relapse and worsening of eating disorders in pregnancy

**DOI:** 10.3389/fpsyg.2025.1601954

**Published:** 2025-04-22

**Authors:** Bente Sommerfeldt, Finn Skårderud, Ingela Lundin Kvalem, Kjersti S. Gulliksen, Arne Holte

**Affiliations:** ^1^Institute of Eating Disorders, Oslo, Norway; ^2^Department of Psychology, Faculty of Social Sciences, University of Oslo, Oslo, Norway; ^3^Faculty of Health Sciences, University of Southern Denmark, Odense, Denmark; ^4^Faculty of Health and Sports Sciences, University of Agder, Kristiansand, Norway; ^5^The Norwegian Psychological Association, Oslo, Norway; ^6^Norwegian Institute of Public Health (NIPH), Oslo, Norway

**Keywords:** eating disorders, anorexia nervosa, bulimia nervosa, pregnancy, relapse, triggers, risk factors

In the published article, there were errors in Table 1. The data in column “Age (Mean 32.8)” should be removed, and the entire column entitled “Number of pregnancies” should be deleted. The corrected table is shown below.

**Table 1:** Details of participants.

	**Age (Mean 32.8)**	Earlier eating disorder diagnosis^*^	Years of treatment for eating disorder^**^	Diagnosis under pregnancy^***^	**Gestations week**	Eating disorder psychopathology (EDE) Global Score (mean 3.8)^****^	**Re-enganged in treatment during pregnancy**
Participant 1		Anorexia nervosa, restrictive type	4	Other specified feeding and eating disorder (OSFED), Atypical Anorexia nervosa	32	4.45	Yes, not specialized
Participant 2		Anorexia nervosa, restrictive type	8	Other specified feeding and eating disorder (OSFED), Atypical Anorexia nervosa	19	2.3	Yes, not specialized
Participant 3		Anorexia nervosa, restrictive type	5	Other specified feeding and eating disorder (OSFED), Atypical Anorexia nervosa	23	5.5	Yes, not specialized
Participant 4		Eating disorder not other specified (EDNOS)	5	Other specified feeding and eating disorder (OSFED), Atypical Anorexia nervosa	16	3.5	Yes, not specialized
Participant 5		Bulimia nervosa	9	Other specified feeding and eating disorder (OSFED), Bulimia nervosa of low frequency	12	3.66	Yes
Participant 6		Anorexia nervosa, restrictive type	15	Other specified feeding and eating disorder (OSFED), Atypical Anorexia nervosa	12	2.7	Yes
Participant 7		Anorexia nervosa, restrictive type	8	None	37	1.5	No
Participant 8		Eating disorder not other specified (EDNOS)	9	Unspecified eating disorder	15	1.78	No
Participant 9		Bulimia nervosa	3	Bulimia Nervosa (BN) -mild	14	4.4	Yes
Participant 10		Anorexia nervosa, restrictive type	17	Other specified feeding and eating disorder (OSFED), Atypical Anorexia nervosa	40	5	Yes, not specialized
Participant 11		Anorexia nervosa, restrictive type	3	Other specified feeding and eating disorder (OSFED), Atypical Anorexia nervosa	9	4.63	Yes, not specialized
Participant 12		Anorexia nervosa, restrictive type	9	Other specified feeding and eating disorder (OSFED), Atypical Anorexia nervosa	25	1.6	Yes, not specialized
Participant 13		Bulimia nervosa	9	Other specified feeding and eating disorder (OSFED), Atypical Anorexia nervosa	22	4.73	Yes, not specialized
Participant 14		Bulimia nervosa	6	Other specified feeding and eating disorder (OSFED), Atypical Anorexia nervosa	16	3.16	Yes
Participant 15		Anorexia nervosa, restrictive type	7	Other specified feeding and eating disorder (OSFED), Atypical Anorexia nervosa)	23	3	Yes
Participant 16		Anorexia nervosa, restrictive type	11	Other specified feeding and eating disorder (OSFED), Bulimia nervosa of low frequency	25	2.44	Yes, not specialized
Participant 17		Bulimia nervosa	3	Bulimia Nervosa (BN) -severe	26	5	Yes, not specialized
Participant 18		Bulimia nervosa	16	Other specified feeding and eating disorder (OSFED), Atypical Anorexia nervosa)	17	5.1	Yes
Participant 19		Anorexia nervosa, binging type	3	Unspecified Eating disorder	16	5.1	Yes, not specialized
Participant 20		Anorexia nervosa, restrictive type	9	Other specified feeding and eating disorder (OSFED), Atypical Anorexia nervosa)	37	3.66	Yes
Participant 21		Bulimia nervosa	7	Other specified feeding and eating disorder (OSFED), Atypical Anorexia nervosa)	32	2.93	Yes, not specialized
Participant 22		Anorexia nervosa, restrictive type	6	Other specified feeding and eating disorder (OSFED), Atypical Anorexia nervosa)	17	3.17	Yes, not specialized
Participant 23		Anorexia nervosa, restrictive type	2	Other specified feeding and eating disorder (OSFED), Atypical Anorexia nervosa)	27	4.4	Yes
Participant 24		Bulimia nervosa	10	Other specified feeding and eating disorder (OSFED), Bulimia nervosa of low frequency	26	5.6	Yes

^*^Earlier diagnosis is self-reported based on DSM-IV diagnosis.

^**^Years of treatment are self-reported by mail after the interview.

^***^Diagnosis based in DSM-5 criteria.

^****^Self-rapport on symptoms using EDE-Q.

Secondly, a correction has been made to **Materials and Methods**, *Data analyses, Paragraph 3*. The incorrect sentence previously stated:

“Each ideal type contains personal features and processed statement observed in at least one, but most often several participants”.

The corrected sentence appears below:

“Each ideal type contains personal features and processed statement observed in several participants.”

Lastly, a correction has been made to **Results**, *The chaotic mother, Paragraph 1*. The incorrect sentence previously stated:

“Britt (41 years) had been diagnosed with bulimia nervosa, severe type.”

The corrected sentence appears below:

“Britt (38 years) had been diagnosed with bulimia nervosa, severe type.”

The authors apologize for these errors and state that they do not change the scientific conclusions of the article in any way. The original article has been updated.

