# Corrigendum: Bodies out of control: relapse and worsening of eating disorders in pregnancy

**DOI:** 10.3389/fpsyg.2025.1621006

**Published:** 2025-05-19

**Authors:** Bente Sommerfeldt, Finn Skårderud, Ingela Lundin Kvalem, Kjersti S. Gulliksen, Arne Holte

**Affiliations:** ^1^Institute of Eating Disorders, Oslo, Norway; ^2^Department of Psychology, Faculty of Social Sciences, University of Oslo, Oslo, Norway; ^3^Faculty of Health Sciences, University of Southern Denmark, Odense, Denmark; ^4^Faculty of Health and Sports Sciences, University of Agder, Kristiansand, Norway; ^5^The Norwegian Psychological Association, Oslo, Norway; ^6^Norwegian Institute of Public Health (NIPH), Oslo, Norway

**Keywords:** eating disorders, anorexia nervosa, bulimia nervosa, pregnancy, relapse, triggers, risk factors

In the published article, there was an error in Table 1 (Details of the participants) as published. Due to concerns about the risk of backward identification, Table 1 has been removed from the article, and the description of the participants has been retained in text form.

Following the removal of Table 1, Table 2 has been renumbered as Table 1. The table previously was labeled as: “Table 2. Ideal types, perceived triggers and personal features.” The corrected Table legend appears below.

“Table 1 Ideal types, perceived triggers and personal features.”

In accordance with the removal of Table 1, corrections have been made to **Results***, Participants*, Paragraphs 1 and 3, and **Results**, *Ideal types*, Paragraph 1.

In **Results**, *Participants*, Paragraph 1, this sentence previously stated.: “Background information, diagnoses and a brief history of the participants' eating disorders are shown in Table 1.” This sentence was removed.

In **Results**, *Participants*, Paragraph 3, this sentence previously stated: “Table 1 shows that 3 qualified for Bulimia Nervosa (BN), 2 for Unspecified Eating Disorder (UFED), 18 for Other Specified Feeding or Eating Disorders (OSFED) and 1 did not qualified for an eating disorder at the time point of interview. OSFED replaces the former Eating Disorder Not Otherwise Specified (EDNOS) category in DSM-IV (American Psychiatric Association, 1994). The table also shows that the symptom pressure measured with EDE-Q Global Score was generally high, varied between 1.6 and 5.6.” The corrected sentence appears below:

“3 qualified for Bulimia Nervosa (BN), 2 for Unspecified Eating Disorder (UFED), 18 for Other Specified Feeding or Eating Disorders (OSFED) and 1 did not qualified for an eating disorder at the time point of interview. OSFED replaces the former Eating Disorder Not Otherwise Specified (EDNOS) category in DSM-IV (American Psychiatric Association, 1994). The symptom pressure measured with EDE-Q Global Score was generally high, varied between 1.6 and 5.6.”

In **Results**, *Ideal types, Paragraph 1*, this sentence previously stated: Table 2 summarize the personal features and perceived triggers in the ideal types”. The corrected sentence appears below:

“Table 1 summarize the personal features and perceived triggers in the ideal types.”

The authors apologize for this error and state that this does not change the scientific conclusions of the article in any way. The original article has been updated.

